# The development and validation of a multidimensional organisational trust measure

**DOI:** 10.3389/fpsyg.2023.1189946

**Published:** 2023-08-14

**Authors:** Sarah Fischer, Arlene Walker, Shannon Hyder

**Affiliations:** Deakin University, Geelong, VIC, Australia

**Keywords:** trust, scale development, multidimensional organisational trust, employee trust in leaders, psychometric validation

## Abstract

**Motivation for the study:**

Workplaces are changing with employees increasingly working remotely and flexibly, which has created larger physical distance between team members. This shift has consequences for trust research and implications for how trust is built and maintained between employees and leaders.

**Research design:**

Three studies collectively aimed to demonstrate how employee trust in leaders has adapted to a hybrid work environment. A validation of a seminal multidimensional employee trust in leaders measure was conducted. Also, an alternative multidimensional measure was developed, piloted, and then validated using exploratory and confirmatory factor analyses.

**Main findings:**

Findings showed the Affective and Cognitive Trust scale not to be sufficiently reliable or valid after testing with a sample working in a hybrid model of virtual and face to face work environments. However, the new measure demonstrated good reliability and validity.

**Implication:**

Findings reinforced that there are behavioural and relational elements to organisational trust, and there are two discreet dimensions to trustworthy behaviour: communication and authenticity.

## Introduction

In 2020, a global pandemic changed the workplace forever. Regardless of organisational policy and culture, employers were forced into flexible and remote working arrangements under the weight of public health orders to reduce or prevent infection with COVID-19 (Spurk and Straub, 2020). As the workplace changed, so did the understanding of how organisational culture was built and maintained (Spicer, 2020). Organisational trust is a key aspect of culture at work (Page et al., 2019). However, during the COVID-19 pandemic, organisational trust was strongly challenged, especially between managers and their employees (Sharieff, 2021).

It is hard to deny that the COVID-19 pandemic has changed the modern workplace globally [Coronavirus: How the world of work may change forever (Internet), 2022]. The main change is the workplace itself. Working from home, having a home that is fit for work, and the technological expectations for working, are key features of that change (Arruda, 2021). One of the significant issues that affected employees was the burden of responsibilities of the home, like childcare and the care of family members who would have engaged in services that closed amidst state and nation-wide lock downs (Chung et al., 2019; Power, 2020) These changes put new stresses on the employee-leader relationship, as expectations for ways of working and performance had to change (Kniffin et al., 2021). This new reality has an impact on organisational trust in the modern workplace (Sharieff, 2021).

### The theory development of multidimensional employee trust in leaders

Organisational trust has been the subject of much research (McEvily and Tortoriello, 2011; Agote et al., 2016) and there is a discourse in the literature regarding whether organisational trust is unidimensional or multidimensional (McAllister, 1995; Dirks and Ferrin, 2002; Schaubroeck et al., 2011). The seminal tool measuring multidimensional employee trust in leaders is McAllister’s (1995) Affective and Cognitive Trust scale (McEvily and Tortoriello, 2011; Fischer et al., 2020). According to McAllister (1995), affective trust is defined as the employee’s emotional connection that forms through a relationship with the leader, while cognitive trust is defined as the employee’s thoughts about the leader’s trustworthy behaviour. Despite its prominence, McAllister’s (1995) scale may not be appropriate for the modern workplace. It has been nearly 30 years since the items have been reviewed for relevancy (Fischer et al., 2020). In addition, the work environment has changed substantially from traditional face-to-face to a blended mode, if not wholly remote working (Graham, 2020; Shine, 2020). A recent meta-analysis (Fischer et al., 2020) found that most of the multidimensional employee trust in leaders research uses McAllister’s (1995) scale. Within the context of great change in how and where work occurs, there is likely to be an impact on employee trust in leaders. Therefore, an updated measure that is reflective of these workplace context changes may be more appropriate.

### Measurement of multidimensional employee trust in leaders

This research presents an in-depth analysis of the reliability and validity of McAllister’s (1995) Affective and Cognitive Trust scale to determine its suitability in the contemporary workplace. There is an important consideration worth exploring in the development of McAllister’s (1995) scale, and thus its construct validity in the contemporary workplace (McEvily and Tortoriello, 2011; Fischer et al., 2020). To design the scale, McAllister (1995) reviewed the available literature and measures of interpersonal trust to devise a list of items. Eleven “organisational behaviour scholars” (p. 35) were employed to review the items against McAllister’s (1995) definitions of affect- and cognition-based trust and classify them accordingly. McAllister (1995) did not provide further clarity about how the definitions of trust were created beyond this in the paper.

It is sound scale development practice to conduct qualitative research to attempt to understand the construct in question before designing the measure (O’Brien, 1993; DeVellis, 2016). There is no overt indication of the use of qualitative research methods to test the content of the items against the real-world work experience of employees trusting their leaders in McAllister’s (1995) scale paper. Contemporary scale development practices consider lack of qualitative research foundations a limitation (Morgado et al., 2017). Even at the time of development of McAllister’s (1995) Affective and Cognitive Trust scale, the use of focus groups was a common practice before developing structured questionnaires (Steckler et al., 1992; O’Brien, 1993). In McAllister’s (1995) original validation study, exploratory factor analysis was used on a data set obtained from a group of postgraduate and undergraduate students to reduce the measure to the 11 items. Although, McAllister (1995) states that the reliability scores for each dimension were good (α = 0.91 and 0.89; p. 36), the selection of participants in 1995 may not reflect the workplace as it cannot be confirmed that students’ experience of employment is generalisable to all employees’ experiences.

### Measurement needs to advance with new context

Given it has been almost 30 years since the Affective and Cognitive Trust scale was first designed, it may not be fully applicable in the contemporary workplace context. It is important to consider the possibility that trust at work may develop and be sustained differently in virtual, hybrid and face-to-face work environments. This is key as technology can mediate interpersonal relationships differently (Carrigan et al., 2020; Bodó, 2021).

A more recent qualitative analysis (Fischer and Walker, 2022) of employees working in a mix of virtual, hybrid and face-to-face work environments showed that employee multidimensional trust in leaders comprised of an explicit behavioural component and an interpersonal relationship component. The findings also discussed the role of communication, exposure, and relationships. These were highlighted as critical in virtual work environments.

Despite the growth of literature exploring how changes in work environment influence interpersonal interaction at work, measurement of multidimensional employee trust in leaders has not.

### Research rationale and hypotheses

The boundary between the home and workplace was becoming a more flexible and fluid environment before the COVID-19 pandemic (Atkinson and Hall, 2011; Galea et al., 2014), but once governments started to impose major restrictions on travel and leaving the home (McLaren and Wang, 2020) the line between home and work virtually disappeared for working adults (Graham, 2020; Power, 2020; Shine, 2020; Arruda, 2021). This change had major impacts on employee relationships and their leader (Kniffin et al., 2021). Based on the considerations highlighted about McAllister’s Affective and Cognitive Trust scale, and the work environment significantly evolving as the world continues to live with the COVID-19 virus and its influence on society, an exploration into the scale’s reliability and validity is warranted. The hypotheses of this research were:

*H1:* McAllister’s (1995) Affective and Cognitive Trust scale, the most often used measure of multidimensional employee trust in leaders in the literature, is less reliable and valid in the contemporary work environment (Study 1).

*H2:* An alternative measure of multidimensional employee trust in leaders, developed using appropriate scale design protocols (Studies 2 and 3), will demonstrate good psychometric properties.

To test these hypotheses, the three studies used a participant sample of the adult population who were working during the COVID-19 pandemic. All three studies were conducted during the COVID-19 pandemic (February–April 2021) when most participants were employed in remote or hybrid working arrangements (Spurk and Straub, 2020), experienced new stresses on the virtual employer–employee working relationship (Power, 2020; Kniffin et al., 2021), and faced major changes to the work environment due to increased infection prevention and control requirements (Mayer, 2021).

## Study 1: Affective and Cognitive Trust Scale Validation

### Method

#### Participants

Study 1 sampled from populations of employed adults working in a range of industries (see Table 1 for detail), with most identifying as aged between 30–39, male, and from health and social assistance fields or professional, scientific and technical services. During April 2021, half of the participants were recruited using nonprobability snowball sampling *via* email and social media through the researcher’s and participants’ communication networks (Leighton et al., 2021; Darko et al., 2022). An advertisement outlined the nature of the study and invited voluntary participation. The remainder of participants were recruited through the online research panel Prolific using convenience sampling. Prolific is a platform to connect academic researchers and participants associated with the University of Oxford (Palan and Schitter, 2018). Consent was provided by submitting the online questionnaire. To ensure the target population for the study, participants were screened by asking if they were currently employed and reported to a manager. The online questionnaire received a total of 251 complete responses.

**Table 1:** Study 1 participant demographics.

	Demographic characteristic	Frequency	Percentage
Gender	Female	81	32.3
	Male	165	65.7
	Prefer not to say	5	2.0
Age	18–24	9	3.6
	25–29	20	8.0
	30–34	77	30.7
	35–39	59	23.5
	40–44	18	7.2
	45–49	16	6.4
	50–54	22	8.8
	55–59	12	4.8
	60–64	18	7.2
Employment type	Full-time	205	81.7
	Part-time	27	10.8
	Contract or contingent worker	19	7.6
Employment length	0–5 years	157	62.5
	6–10 years	42	16.7
	11–15 years	18	7.2
	16–25 years	12	4.8
	26–40 years	20	8.0
	40+ years	2	0.8
Industry	Construction	2	0.8
	Retail trade	9	3.6
	Information media and telecommunications	6	2.4
	Rental, hiring and real estate services	8	3.2
	Professional, scientific and technical services	69	27.5
	Administrative and support services	9	3.6
	Public administration and safety	11	4.4
	Education and training	33	13.1
	Health care and social assistance	80	31.9
	Other services	24	9.6

*N* = 251.

#### Data handling

Data were screened through IBM SPSS Statistics Version 27 (SPSS; IBM Corp, 2020) to assess accuracy of input, missing values, univariate outliers, linearity, and normality. No skew or kurtosis was identified and there was no missing data.

#### Analysis

To assess the internal consistency of the items and subscales of McAllister’s (1995) scale, item analysis was conducted using the following process, recommended by DeVellis (2003), Fishman and Galguera (2003), Kline (1999), Spector (1992), and Walker (2010). The negatively phrased items in McAllister’s (1995) scale were reverse scored to ensure positive correlations. Descriptive statistics were then reviewed. Items would be considered as contributing little to internal consistency if they showed a low item-total correlation coefficient (< 0.60), a large standard deviation (> 0.30), or a large skew index (> 6.00; Spector, 1992; Kline, 1999; DeVellis, 2003; Fishman and Galguera, 2003; Walker, 2010).

Confirmatory Factor Analysis was performed next using SPSS AMOS Version 27 (AMOS; IBM Corp, 2020) to determine if the McAllister’s (1995) items and subscales were a good fit to the scale model. This analysis was conducted to determine if the scale’s factor structure holds within the context of the sample population of working adults during the COVID-19 pandemic. This analysis involved proposing a set of relationships and evaluating the consistency of this model in an observed covariance-matrix (Hu and Bentler, 1999). Interpretation of the model fit indices used in this analysis are shown in Table 2.

**Table 2:** Confirmatory factor analysis model fitness indicators interpretation.

Fit indicator	Interpretation
Maximum likelihood chi-square estimation (chi-square estimation)	Significant chi-square value indicates model fit and non-significant chi-square value indicates lack of model fit (Tabachnick and Fidell, 2011). Sensitive to sample size (Rolke and Gongora, 2021). Tabachnick and Fidell (2011) recommend the use of a normed chi-square statistic (χ^2^/df) because it accounts for model complexity, and values between one and three indicate a satisfactory fit, with a value less than two indicating a good fit.
Root mean standardized error of approximation (RMSEA)	Absolute fit index. Used to determine the extent to which the proposed model predicts the sample data. One of the most informative indices of model fit. Considers the error of approximation in the population and is sensitive to the number of parameters in the model (Xia and Yang, 2019). Values for the RMSEA should be less than 0.08 to suggest a reasonable fit and less than 0.05 for a good fit to the model (Xia and Yang, 2019).
Standardized root mean square residual (SRMR)	Absolute fit index. Used to determine the extent to which the proposed model predicts the sample data. Hu and Bentler (1999) recommend using SRMR with the RMSEA as it evaluates model fit by evaluating residual matrices. Values for SRMR should be less than 0.06 to suggest a good-fitting model (Hu and Bentler, 1999).
Normed fit index (NFI), comparative fit index (CFI), relative fit index (RFI) and Tucker Lewis index (TLI)	Incremental fit indices compare the proposed model to the null model where the variables are specified not to correlate. Indices range from zero to one, with values above 0.90 representing acceptable fit and greater than 0.95 regarded as a good fit. Incremental fit indices can be used together to offer additional consideration about model fit (Hu and Bentler, 1999) but should not be relied on without considering absolute fit indices (Kenny et al., 2015).

### Results

#### Internal consistency reliability

The 11-item Affective and Cognitive Trust scale (McAllister, 1995) was separated into its affective (items 1–5) and cognitive (items 6–11) subscales to conduct reliability analyses. The reliability analyses showed that both subscales had high Cronbach alpha scores (affective trust α = 0.89; cognitive trust α = 0.91). However, examining at the item-level for each subscale showed that three of the items (items 3, 5 and 11 – see Table 3) had low inter-item correlations, well below what would be considered acceptable for a well-validated scale (DeVellis, 2016; < 0.65). Many of the items (2, 4, 7, 9, and 10) also suggested multicollinearity (Grewal et al., 2004) inter-item total correlations >0.80. These findings in total suggest that the scale has less than optimal internal consistency. Meaning, although the subscales appeared internally consistent, the item analyses indicated that there were some unnecessary and some redundant items for this sample.

**Table 3:** Item analysis of McAllister’s (1995) affective and cognitive trust subscales.

Subscale	Item	*M*	*SD*	Skew	Item-total correl.	Sq mult. *R*	*α* if deleted
Affective trust	1. We have a sharing relationship. We can both freely share our ideas, feelings, and hopes.	2.99	1.331	0.081	0.785	0.765	0.928
	2. I can talk freely to this individual about difficulties I am having at work and know that (s)he will want to listen.	3.00	1.414	−0.037	0.828	0.778	0.926
	3. We would both feel a sense of loss if one of us was transferred and we could no longer work together.	2.88	1.310	0.152	0.512	0.495	0.939
	4. If I shared my problems with this person, I know (s)he would respond constructively and caringly.	3.03	1.381	−0.223	0.841	0.772	0.925
	5. I would have to say that we have both made considerable emotional investments in our working relationship.	3.06	1.122	−0.044	0.589	0.566	0.936
Cognitive trust	6. This person approaches his/her job with professionalism and dedication.	2.87	1.472	0.107	0.786	0.775	0.928
	7. Given this person’s track record, I see no reason to doubt his/her competence and preparation for the job.	2.89	1.489	−0.076	0.847	0.815	0.925
	8. I can rely on this person not to make my job more difficult by careless work.	3.01	1.362	−0.179	0.663	0.538	0.933
	9. Most people, even those who aren’t close friends of this individual, trust and respect him/her as a co-worker.	2.98	1.262	−0.199	0.820	0.794	0.927
	10. Other work associates of mine who must interact with this individual consider him/her to be trustworthy.	3.18	1.300	−0.334	0.821	0.771	0.927
	11. If people knew more about this individual and his/her background, they would be more concerned and monitor his/her performance more closely^a^.	2.55	1.313	0.462	0.535	0.386	0.938

a Reverse scored item.

#### Confirmatory factor analysis

Confirmatory factor analysis was employed to confirm the item structure of the Affective and Cognitive Trust scale (McAllister, 1995). Despite indications from the internal consistency analysis that some items were unnecessary, all items were retained in their original factor structure in the confirmatory factor analysis to further examine whether McAllister’s (1995) scale is valid in the contemporary work environment. The analysis revealed that the data was not an optimal fit to the model χ^2^ (43, *N* = 255) = 239.50, *p* > 0.001; PCMIN/DF = 5.57; NFI = 0.90; CFI = 0.92; RFI = 0.87; TLI = 0.89; RMSEA = 0.14 *and* RMSEA confidence interval = 0.12–0.15; SRMR = 0.06. Although CFI was acceptable, the NFI, RFI, TLI, and RMSEA indices fell below the criterion for a satisfactory fit. The NFI, RFI, and TLI violations suggested this model was not better than the null model (Hu and Bentler, 1999; Kenny et al., 2015), whereas the outcomes of RMSEA suggested this model was different from the perfect model (Hu and Bentler, 1999; Xia and Yang, 2019). Although the purpose of this analysis was to test the current model’s validity, an attempt was made to improve fit by changing the model based on the outcomes of the modification indices test using AMOS. Two affective items appeared to be predicted by the cognitive trust factor with this sample. Those items were moved from the affective trust factor to cognitive trust, but the model did not improve. Therefore, the model was deemed not fit for the sample and *H1* was supported.

## Study 2: alternative measure pilot

Study 2 aimed to determine if an alternative multidimensional employee trust in leaders scale could be designed that was reliable and valid for the contemporary work environment.

### Item development

Items were developed based on a qualitative study on Australian corporate employees’ experience trusting their director managers and senior leaders (Fischer and Walker, 2022). The study was conducted to explore trust in traditional face-to-face and virtual work environments and used critical incident technique (Flanagan, 1954; Dugar, 2018). Qualitative research methods (Braun and Clarke, 2020, 2021) using real-world examples enabled greater clarity of the individual behaviours that constitute multidimensional employee trust in leaders, thereby ensuring the measure’s potential items were relevant for the contemporary workplace.

The following guidelines were observed in generating the items (Spector, 1992; Kline, 1999; DeVellis, 2016):

1. All items were written in brief, plain English (e.g., no jargon or colloquialisms).
2. No items were written in a double-barrelled manner, so to communicate easily to the respondent what they are meant to rate.
3. As much as possible, negatively phrased items were avoided.

Likert-type scales of agreement are useful in behavioural research (DeVellis, 2016), where items are presented as statements and participants are asked their level of agreement with each statement. The literature suggests that five-point scales are useful to increase response rate (Babakus and Mangold, 1992; Hayes, 1992; Devlin et al., 2003), response reliability and variability (Lissitz and Green, 1975; McKelvie, 1978; Jenkins and Taber, 1997). The verbal anchors were displayed going from the positive “Strongly agree” and ending with the negative “Strongly disagree”, which is the preferred method (Sheluga et al., 1978; Yan and Keusch, 2015). Items for the new scale were reviewed by two experts with experience in psychometrics and scale development before finalising the measure for the Alternative Measure Pilot study.

### Method

#### Participants

Pilot Study participants were employed adults with a manager to whom they reported. The ideal number of participants for pilot testing measures ranges from 75 to 200 (Hertzog, 2008; Johanson and Brooks, 2010). The sample in this pilot study was *N* = 118, and therefore considered acceptable (Thompson, 2004). Participant demographics are shown in Table 4, with most aged 30–39, identifying as female, and from health and social assistance fields or professional, scientific and technical services.

**Table 4:** Study 2 participant demographics.

	Demographic characteristic	Frequency	Percentage
Gender	Female	82	69.5
	Male	34	28.8
	Gender non-specified	2	1.7
Age	18–24	4	3.4
	25–29	9	7.6
	30–34	36	30.5
	35–39	26	22.0
	40–44	9	7.6
	45–49	8	6.8
	50–54	11	9.3
	55–59	6	5.1
	60–64	9	7.6
Employment type	Full-time	96	81.4
	Part-time	13	11.0
	Contract or contingent worker	9	7.6
Employment length	0–5 years	73	61.9
	6–10 years	19	16.1
	11–15 years	9	7.6
	16–25 years	6	5.1
	26–40 years	10	8.5
	40+ years	1	0.8
Industry	Construction	1	0.8
	Retail trade	4	3.4
	Information media and telecommunications	3	2.5
	Rental, hiring and real estate services	3	2.5
	Professional, scientific, and technical services	33	28.0
	Administrative and support services	4	3.4
	Public administration and safety	5	4.2
	Education and training	16	13.6
	Health care and social assistance	38	32.2
	Other services	11	9.3

*N* = 118.

#### Procedure

During February 2021, participants were recruited using nonprobability snowball sampling *via* email and social media through the researcher’s and participants’ communication networks (Leighton et al., 2021; Darko et al., 2022). An advertisement outlined the nature of the study and invited voluntary participation. Consent was provided by submitting the online questionnaire. Participants were screened by asking if they were currently employed and reported to a manager.

#### Measure

The developed measure contained 49 items generated from a review of the literature and a qualitative study on multidimensional employee trust in leaders (Fischer and Walker, 2022). Table 5 illustrates the type of trustworthy behaviours and facets of relationship quality explored in this new measure of organisational trust with an example item for each.

**Table 5:** Example items measuring multidimensional employee trust in leaders.

Theme	Subtheme	Example item
Trustworthy behaviour	Honesty and integrity	I believe my manager has integrity
	Communication behaviours	My manager shares information with me regularly
	Reliability	My manager does what they say they will do
	Support	My manager supports me when I need it
	Equality and respect	My manager treats me as an equal
Relationship quality	Exposure	My manager and I interact regularly
	Rapport and understanding	I feel that I know my manager well
	Sameness	I can relate to my manager because we have something in common
	Leader vulnerability	My manager acknowledges that at times I have strength where they have weakness
	Employee psychological safety	I feel safe to take risks under my manager’s leadership
	Emotional connection	I would feel sad if my manager was no longer in the role

#### Analysis

A process recommended by DeVellis (2003), Fishman and Galguera (2003), Kline (1999), Spector (1992), and Walker (2010) was followed to determine item and scale reliability. Negatively phrased items were initially reverse scored to ensure positive correlations. Descriptive statistics and item analyses were conducted to refine the multidimensional employee trust in leaders scale. This process involved inspecting item means and standard deviations to identify floor or ceiling effects and to ensure a range in responses; skew indices, corrected item-total correlations, squared multiple correlations, and α if item deleted were also examined. Items were considered for deletion if they met at least one of the following criteria: a low item-total correlation coefficient (< 0.60), a large standard deviation (> 0.30), or a large standardised skew index (> 6.0). Finally, a review of the items was carried out to identify ambiguous or redundant items.

Exploratory factor analysis was conducted to investigate underlying constructs using principal component analysis with varimax rotation. Principal component analysis was selected because the goal of the analysis was to reduce the number of items and determine which items were most salient to the final scale and subscales (Tabachnick and Fidell, 2011). Varimax was selected as it is a popular orthogonal rotation method used in Exploratory Factor Analysis (Tabachnick and Fidell, 2007). Finally, reliability analyses using Cronbach’s alpha was conducted to determine internal consistency.

### Results

Table 6 presents the descriptive statistics and item analysis for the 49 items comprising the multidimensional employee trust in leaders scale. An examination of the descriptive statistics and item analyses identified item 29 for deletion from the scale due to its extremely low item correlation. At the conclusion of the item analysis, the scale retained 48 items and Cronbach’s alpha indicated high internal consistency (α = 0.98).

**Table 6:** Descriptive statistics, item analysis, and reworded items for multidimensional employee trust in leaders scale.

Item	*M*	*SD*	Stand. skew	Item-total correl.	Squared *r*.	*α* if deleted
1. I believe my manager has integrity	1.90	1.150	5.363	0.824	0.679	0.980
2. My manager is honest	1.81	1.015	5.381	0.687	0.472	0.980
3. My manager does not lie	2.19	1.207	3.202	0.666	0.444	0.980
4. My manager shares information with me regularly	2.18	1.174	4.632	0.777	0.604	0.980
5. My manager regularly communicates with me	2.08	1.195	4.735	0.765	0.585	0.980
6. My manager tells me what I need to know to keep me informed	2.22	1.192	3.565	0.808	0.653	0.980
7. My manager empathises with me when I feel challenged	2.25	1.276	3.771	0.798	0.637	0.980
8. I feel I can share my frustrations openly to my manager	2.38	1.280	3.099	0.748	0.560	0.980
9. My manager listens to me	2.11	1.182	4.404	0.862	0.743	0.980
10. My manager is consistent with their communication	2.38	1.346	3.265	0.748	0.560	0.980
11. My manager does what they say they will do	2.19	1.240	4.022	0.724	0.524	0.980
12. My manager says one thing and does another^a^	3.58	1.303	2.592	0.559	0.312	0.983
13. I believe that I can tell my manager something and it will not be repeated without my permission	2.57	1.459	1.874	0.768	0.590	0.980
14. The information I share with my manager is kept confidential	2.42	1.336	2.610	0.727	0.529	0.980
15. My manager can keep a secret	2.48	1.363	2.103	0.689	0.475	0.980
16. I do not believe my manager keeps information from me	2.92	1.343	0.126	0.667	0.445	0.980
17. My manager communicates transparently	2.50	1.345	2.839	0.874	0.764	0.980
18. My manager communicates openly	2.36	1.350	3.072	0.896	0.803	0.980
19. My manager supports me when I need it	2.03	1.240	5.108	0.845	0.714	0.980
20. My manager supports me when I have made a mistake	2.05	1.139	4.457	0.800	0.640	0.980
21. My manager backs me up in front of others	2.13	1.144	4.022	0.772	0.596	0.980
22. My manager goes above and beyond to support me	2.58	1.250	2.381	0.814	0.663	0.980
23. My manager treats me as an equal	2.42	1.464	2.735	0.860	0.740	0.980
24. My manager respects me	1.89	1.108	5.296	0.853	0.728	0.980
25. My manager includes me in decisions	2.47	1.266	3.202	0.804	0.646	0.980
26. My manager regards treats me as inferior^a^	3.75	1.391	3.206	0.625	0.391	0.984
27. My manager and I interact regularly	2.03	1.240	5.108	0.643	0.413	0.980
28. My manager and I spend time together	2.50	1.273	2.157	0.584	0.341	0.981
29. I have known my manager a long time	3.02	1.371	0.224	0.250	0.063	0.981
30. My manager and I have a good rapport	1.94	1.127	5.435	0.861	0.741	0.980
31. I feel that I know my manager well	2.26	1.136	3.834	0.704	0.496	0.980
32. My manager and I have built a relationship	2.03	1.090	5.919	0.794	0.630	0.980
33. My manager and I are similar	3.35	1.290	−0.852	0.698	0.487	0.980
34. I can relate to my manager because we have something in common	2.75	1.255	1.390	0.741	0.549	0.980
35. My manager and I have shared interests or values	2.52	1.204	3.444	0.764	0.584	0.980
36. My manager has been vulnerable in front of me	2.62	1.395	2.587	0.661	0.437	0.980
37. My manager is not afraid to be vulnerable around me	2.51	1.266	2.395	0.680	0.462	0.980
38. My manager acknowledges that at times I have strength where they have weakness	2.35	1.434	3.341	0.915	0.837	0.980
39. I believe that I can trust my manager	2.36	1.423	3.480	0.895	0.801	0.980
40. I have confidence that my manager can be trusted	2.57	1.399	2.099	0.906	0.821	0.980
41. I feel safe to take risks under my manager’s leadership	2.61	1.365	1.933	0.861	0.741	0.980
42. My manager makes me feel secure	2.61	1.371	2.121	0.830	0.689	0.980
43. I am protected by my manager	1.79	1.003	6.139	0.794	0.630	0.980
44. My manager trusts me	2.50	1.259	2.224	0.753	0.567	0.980
45. I trust my manager because they trust me	2.21	1.280	3.309	0.905	0.819	0.980
46. Trust is reciprocated between my manager and I	2.78	1.372	1.283	0.849	0.721	0.980
47. I feel an attachment to my manager	1.91	1.078	5.883	0.690	0.476	0.980
48. I care about my manager as a person	2.46	1.394	2.430	0.823	0.677	0.980
49. I would feel sad if my manager was no longer in the role	2.51	1.204	2.996	0.828	0.686	0.980

a Reverse scored item.

Factor analysis with varimax rotation was carried out on the remaining 48-items. Factorability was established by examining the correlation matrix, the Kaiser-Meyer-Olkin Measure of Sampling Adequacy (KMO) and Bartlett’s Test of Sphericit (Browne, 2001). The correlation matrix revealed that all correlations were more than the recommended 0.30; the obtained KMO value was more than the minimal 0.60 at 0.951; and Bartlett’s Test of Sphericity was significant (*p* < 0.001), thus indicating a data set suitable for factor analysis.

A principal components extraction was used to produce the initial unrotated solution for the scale. Five Eigenvalues greater than one accounted for 74.7% of the variance. The scree plot also indicated the presence of five factors. The following criteria (Browne, 2001; Sellbom and Tellegen, 2019) were used to determine the items that load onto a factor and the stability of a factor: (1) An assigned value of >0.40 was used to identify items with substantive loadings on a factor. (2) Items with loadings >0.40 were also required to load at least 0.20 less on other factors to be considered as distinctive items. Items loading onto more than one factor were included in the factor with the highest loading if the items were distinctive. Items loading onto more than one factor that were not distinctive were not included in any factor. (3) The stability of a factor was determined by the factor having at least three items loading onto it both substantively and distinctively. The rotated factor structure did not yield distinguishable factors. Many items had cross-loadings that were not distinctive, and the factors lacked stability. Therefore, based on the results of Study 2, the wording of several items (6, 8, 19, 26, 31, 32, 36, 41, 47, 49) was revised to determine clearer differences and definitions of factors.

## Study 3: alternative measure preliminary validation

Given the reliability and overall model fit problems seen in Study 1’s examination of McAllister’s (1995) Affective and Cognitive Trust scale, Study 3 was run on the revised battery of items from Study 2 on a new sample to address the second hypothesis.

### Method

#### Participants

As with Studies 1 and 2, the sample consisted of employed adults with a manager to whom they reported. Participant demographics are shown in Table 7. The participant demographic profile for this sample was more evenly split across the genders than the samples from Studies 1 and 2. There was a greater skew to the younger ages of 18–34. Whilst the industry range was more even, there remained a greater number of health and social assistance participants.

**Table 7:** Study 3 participant demographics.

	Demographic characteristic	Frequency	Percentage
Gender	Female	250	52.3
	Male	224	46.9
	Gender non-specified	4	0.8
Age	18–24	169	35.4
	25–29	101	21.1
	30–34	74	15.5
	35–39	37	7.7
	40–44	22	4.6
	45–49	25	5.2
	50–54	20	4.2
	55–59	12	2.5
	Over 60	18	3.7
Employment type	Full-time	345	72.2
	Part-time	121	25.2
	Contract or contingent worker	12	2.5
Employment length	0–5 years	327	68.4
	6–10 years	66	13.8
	11–15 years	40	8.4
	16–25 years	27	5.6
	26–40 years	14	2.9
	40+ years	4	0.8
Industry	Agriculture	5	1.0
	Mining	2	0.4
	Manufacturing	29	6.1
	Electricity, gas, water, and waste services	6	1.3
	Construction	7	1.5
	Wholesale trade	3	0.6
	Retail trade	27	5.6
	Accommodation and food services	24	5.0
	Transport, postal and warehousing	15	3.1
	Information media and telecommunications	43	9.0
	Professional, scientific and technical services	58	12.1
	Administrative and support services	34	7.1
	Public administration and safety	26	5.4
	Education and training	33	6.9
	Health care and social assistance	112	23.4
	Arts and recreation services	10	2.1
	Other services	44	9.2

*N* = 485, seven cases did not include demographic data.

#### Procedure

Participant recruitment was conducted in two phases. First, in March 2021 *via* email and social media through the researcher’s and participants’ communication networks using nonprobability snowball sampling (Leighton et al., 2021; Darko et al., 2022) Second, in April 2021 *via* a research panel *via* the data collection agency Prolific using convenience sampling. All participants were screened to ensure they were currently employed and reported to a manager. Thirty-three cases were removed due to substantial missing data, with the remaining number of participants being 485.

#### Measure

The 48 items identified and revised from Study 2 was used to measure employee multidimensional employee trust in leaders (see Table 6).

#### Data handling

Data were screened through IBM SPSS Statistics Version 27 (SPSS; IBM Corp, 2020) to assess accuracy of input, missing values, univariate outliers, linearity, and normality. No skew or kurtosis was identified and there was no missing data.

To run both planned analyses for Study 3, the full data set (*N* = 485) was randomly split into two data sets using IBM SPSS Statistics Version 27 (SPSS; IBM Corp, 2020). A set of cases (*N* = 241) were analysed using exploratory factor analysis. The remainder of the cases (*N* = 244) was used for the confirmatory factor analysis.

#### Analysis

The same process used in the pilot study was used in the validation study to determine item and scale reliability. The sample of *N* = 241 was considered sound for exploratory factor analysis (Thompson, 2004). Items were again considered for deletion from the scales if they met at least one of the following criteria: a low item-total correlation coefficient (< 0.60), a large standard deviation (> 0.30), or a large standardised skew index (> 6.00; Spector, 1992; Kline, 1999; DeVellis, 2003; Fishman and Galguera, 2003; Walker, 2010).

Exploratory factor analysis was used to determine the factor structure of the scale using principal component analysis. Direct oblimin rotation was selected based on the high number of items and high internal consistency, being a preferred rotation method when variables are correlated (Kline, 2014). Reliability analyses using Cronbach’s alpha was used to determine internal consistency of the subscales.

Confirmatory factor analysis was undertaken following the exploratory factor analysis to determine if the factor structure observed could be confirmed in an equivalent sample. The sample size (*N* = 244) was considered sound for confirmatory factor analysis (Thompson, 2004). This analysis involved proposing a set of relationships and evaluating the consistency of this model in an observed covariance-matrix (Hu and Bentler, 1999). Interpretation of the model fit indices used in this analysis can be found in Table 2.

### Results

#### Item analysis

Table 8 presents the descriptive statistics and item analysis prior to conducting exploratory factor analysis with the 48 items comprising the organisational trust scale. An examination of the descriptive statistics and item analyses identified six items (8, 11, 19, 24, 38, and 40) for deletion from the trust scale based on low item-total correlations (0.65). High skewness was originally included in the analysis to identify items for deletion; however, no items were selected for deletion based on skewness alone. This choice was made because participants responses may have been influenced by the drastic global experience of forced virtual work environments (Kaushik and Guleria, 2020; Trougakos et al., 2020) as the data was collected during the height of the COVID-19 pandemic.

**Table 8:** Descriptive statistics and item analysis for multidimensional employee trust in leaders scale.

Item	*M*	*SD*	Stand. skew	Item-total correl.	Square mult *R*.	*α* if deleted
1. The information I share with my manager is kept confidential	2.28	1.124	4.305	0.709	0.503	0.977
2. My manager regularly communicates with me	2.05	1.113	6.656	0.696	0.484	0.977
3. My manager is honest	2.20	1.131	5.740	0.804	0.646	0.977
4. I would feel sad if my manager left	2.57	1.322	2.961	0.805	0.648	0.977
5. I believe my manager has integrity	2.16	1.128	5.669	0.790	0.624	0.977
6. My manager makes me feel secure	2.43	1.215	3.370	0.834	0.696	0.977
7. I have confidence that my manager can be trusted	2.26	1.218	4.942	0.841	0.707	0.977
8. I have seen my manager be vulnerable	2.84	1.316	1.195	0.381	0.145	0.978
9. My manager trusts me	1.95	0.923	7.487	0.707	0.500	0.977
10. I am protected by my manager	2.51	1.162	3.273	0.782	0.612	0.977
11. I know my manager well	2.54	1.130	2.299	0.586	0.343	0.978
12. My manager communicates openly	2.19	1.184	6.110	0.795	0.632	0.977
13. Trust is reciprocated between my manager and I	2.24	1.122	5.422	0.848	0.719	0.977
14. My manager tells me what I need to know	2.03	1.098	8.136	0.739	0.546	0.977
15. My manager respects me	1.96	1.041	7.351	0.748	0.560	0.977
16. My manager supports me	2.12	1.081	6.890	0.847	0.717	0.977
17. I believe that I can tell my manager something and it will not be repeated without my permission	2.44	1.253	3.338	0.778	0.605	0.977
18. My manager and I interact regularly	2.12	1.054	6.182	0.644	0.415	0.978
19. My manager treats me as inferior^a^	3.55	1.250	2.955	0.513	0.263	0.981
20. My manager does what they say they will do	2.32	1.129	5.071	0.751	0.564	0.977
21. I trust my manager because they trust me	2.46	1.160	3.851	0.785	0.616	0.977
22. My manager empathises with me when I feel challenged	2.52	1.202	3.494	0.766	0.587	0.977
23. My manager includes me in decisions	2.55	1.224	4.403	0.723	0.523	0.977
24. My manager and I spend time together	3.14	1.308	−0.006	0.570	0.325	0.978
25. My manager and I have a good rapport	2.17	1.035	5.864	0.811	0.658	0.977
26. My manager backs me up in front of others	2.48	1.111	2.838	0.775	0.601	0.977
27. My manager goes above and beyond to support me	2.81	1.198	1.571	0.803	0.645	0.977
28. My manager shares information with me regularly	2.29	1.149	5.747	0.735	0.540	0.977
29. My manager can keep a secret	2.47	1.235	3.591	0.734	0.539	0.977
30. My manager supports me when I have made a mistake	2.35	1.123	4.422	0.749	0.561	0.977
31. My manager treats me as an equal	2.42	1.242	4.169	0.755	0.570	0.977
32. My manager listens to me	2.13	1.060	6.643	0.832	0.692	0.977
33. I feel a connection with my manager	2.75	1.284	2.182	0.821	0.674	0.977
34. I can share my inner thoughts openly to my manager	2.84	1.268	1.935	0.759	0.576	0.977
35. I can relate to my manager because we have something in common	2.77	1.185	2.136	0.739	0.546	0.977
36. My manager and I are similar	3.10	1.227	−0.019	0.665	0.442	0.977
37. My manager acknowledges that at times I have strength where they have weakness	2.66	1.174	2.669	0.727	0.529	0.977
38. I do not believe my manager keeps information from me	2.82	1.196	1.649	0.593	0.352	0.978
39. My manager is not afraid to be vulnerable around me	2.96	1.213	0.805	0.663	0.440	0.977
40. My manager says one thing and does another^a^	3.47	1.317	3.123	0.528	0.279	0.981
41. I believe that I can trust my manager	2.35	1.188	5.201	0.848	0.719	0.977
42. My manager does not lie	2.75	1.173	1.799	0.693	0.480	0.977
43. I have a good relationship with my manager	2.17	1.091	6.723	0.821	0.674	0.977
44. I care about my manager as a person	2.20	1.151	6.058	0.758	0.575	0.977
45. My manager is consistent with their communication	2.38	1.208	4.968	0.803	0.645	0.977
46. My manager communicates transparently	2.30	1.214	5.561	0.821	0.674	0.977
47. Under my manager I feel safe to take risks	2.66	1.253	3.155	0.767	0.588	0.977
48. My manager and I have shared interests or values	2.59	1.184	2.955	0.778	0.605	0.977

*N* = 241.

a Reverse scored item.

#### Exploratory factor analysis

Exploratory factor analyses using principal component analysis with direct oblimin rotation was carried out on the remaining 42 items (Kline, 2014). Direct oblimin rotation was selected due to the high number of items and high internal consistency as it is a preferred rotation method when variables are correlated (Browne, 2001). Factorability of each data set was established by examining the correlation matrix, the Kaiser-Meyer-Olkin Measure of Sampling Adequacy (KMO) and Bartlett’s Test of Sphericity (Browne, 2001). The correlation matrix revealed that all correlations were above the recommended 0.30; the obtained KMO value exceeded the minimal 0.60 at 0.97 (Kaiser, 1970; Kaiser, 1974); and Bartlett’s Test of Sphericity was significant (Bartlett, 1954) (*p* < 0.001), thus indicating a data set suitable for factor analysis.

A principal components extraction was used to produce the initial unrotated solution for the scale. Three Eigenvalues greater than one accounted for 66.3% of the variance. The scree plot also indicated the presence of three factors. The pattern matrix, produced from the direct oblimin rotation, was explored to identify which items loaded onto the three factors (Kline, 2014). The same criteria as used in the Pilot Study determined the items that loaded onto a factor and the stability of each factor.

Table 9 shows the three distinguishable factors that emerged, as well as the items associated with each factor. These factors were labelled Authentic Behaviours (F1), Interpersonal Connection and Care (F2), and Consistent Communication (F3). The rationale for the names ascribed to each factor was based on the nature of the items that emerged and the discussion of behavioural and relational elements to multidimensional employee trust in leaders (Dirks and Ferrin, 2002; Schoorman et al., 2007; McEvily and Tortoriello, 2011). Reliability analysis showed strong Cronbach alphas (Authentic Behaviours α = 0.94; Interpersonal Connection and Care α = 0.87; Consistent Communication α = 0.83).

**Table 9:** Rotated factor structure of the scale.

Item	Factor Eigenvalue % variance	F1 25.001 59.53%	F2 1.522 3.62%	F3 1.327 3.16%
1 – The information I share with my manager is kept confidential		0.924		
3 – My manager is honest		0.865		
6 – My manager makes me feel secure		0.840		
7 – I have confidence that my manager can be trusted		0.859		
13 – Trust is reciprocated between my manager and I		0.763		
15 – My manager respects me		0.718		
20 – My manager does what they say they will do		0.751		
34 – I can share my inner thoughts openly to my manager			0.535	
35 – I can relate to my manager because we have something in common			0.731	
36 – My manager and I are similar			0.823	
39 – My manager is not afraid to be vulnerable around me			0.719	
48 – My manager and I have shared interests or values			0.542	
2 – My manager regularly communicates with me				0.720
18 – My manager and I interact regularly				0.747
28 – My manager shares information with me regularly				0.612

*N* = 241.

#### Confirmatory factor analysis

Finally, confirmatory factor analysis was conducted (*N* = 244) and all items’ correlation values were in an acceptable range (Tabachnick and Fidell, 2011) of ≥0.30 and ≤ 0.80. See Table 10 for the item descriptives and correlations and Table 11 for descriptive statistics, reliability scores and correlations of the factors.

**Table 10:** Means, SD, and correlations between items retained in the structure.

	Mean	SD	EMT1	EMT3	EMT6	EMT7	EMT1	EMT1	EMT2	EMT3	EMT3	EMT3	EMT3	EMT4	EMT2	EMT1	EMT28
EMT1	2.21	1.079	–														
EMT3	2.08	1.048	0.597^***^	–													
EMT6	2.23	1.126	0.633^***^	0.768^***^	–												
EMT7	2.16	1.107	0.600^***^	0.790^***^	0.746^***^	–											
EMT13	2.14	1.100	0.601^***^	0.760^***^	0.799^***^	0.783^***^	–										
EMT15	1.86	0.923	0.510^***^	0.649^***^	0.663^***^	0.641^***^	0.749^***^	–									
EMT20	2.20	1.044	0.481^***^	0.690^***^	0.667^***^	0.741^***^	0.676^***^	0.551^***^	–								
EMT34	2.70	1.243	0.426^***^	0.607^***^	0.594^***^	0.653^***^	0.658^***^	0.541^***^	0.543^***^	–							
EMT35	2.61	1.135	0.409^***^	0.544^***^	0.545^***^	0.553^***^	0.593^***^	0.534^***^	0.507^***^	0.626^***^	–						
EMT36	2.95	1.215	0.361^***^	0.412^***^	0.432^***^	0.437^***^	0.517^***^	0.398^***^	0.383^***^	0.533^***^	0.671^***^	–					
EMT39	2.92	1.125	0.377^***^	0.412^***^	0.442^***^	0.459^***^	0.494^***^	0.391^***^	0.382^***^	0.516^***^	0.518^***^	0.436^***^	–				
EMT48	2.47	1.140	0.401^***^	0.618^***^	0.637^***^	0.615^***^	0.656^***^	0.576^***^	0.584^***^	0.592^***^	0.701^***^	0.608^***^	0.497^***^	–			
EMT2	2.00	1.029	0.401^***^	0.553^***^	0.579^***^	0.556^***^	0.534^***^	0.426^***^	0.477^***^	0.518^***^	0.439^***^	0.260^***^	0.375^***^	0.494^***^	–		
EMT18	2.05	1.032	0.290^***^	0.447^***^	0.435^***^	0.526^***^	0.515^***^	0.449^***^	0.467^***^	0.473^***^	0.440^***^	0.338^***^	0.363^***^	0.480^***^	0.660^***^	–	
EMT28	2.27	1.067	0.408^***^	0.518^***^	0.522^***^	0.526^***^	0.555^***^	0.456^***^	0.585^***^	0.510^***^	0.529^***^	0.394^***^	0.410^***^	0.538^***^	0.596^***^	0.586^***^	–

*N* = 244, ***p < 0.001.

**Table 11:** Means, SDs, Cronbach alphas and correlations for the three factors.

Variable	Mean	SD	*α*	1	2	3
1. Authentic behaviour	2.17	0.94	0.93	–		
2. Interpersonal connection and care	2.81	1.00	0.88	0.749***	–	
3. Consistent communication	2.13	0.952	0.83	0.657***	0.667***	–

*N* = 244, ***p < 0.001.

To ensure comparability between the two subsamples of the original data set used for the exploratory factor analysis and the confirmatory factor analysis, a series of one-way ANOVAs and chi-square tests were conducted. The ANOVAs and chi-square tests were based on the factors identified in the exploratory factor analysis (see Table 9: F1, F2 and F3), Age and Gender. No significant differences between the sample used for the exploratory factor analysis and the sample used for the confirmatory factor analysis were found for any variable.

**Figure 1:**
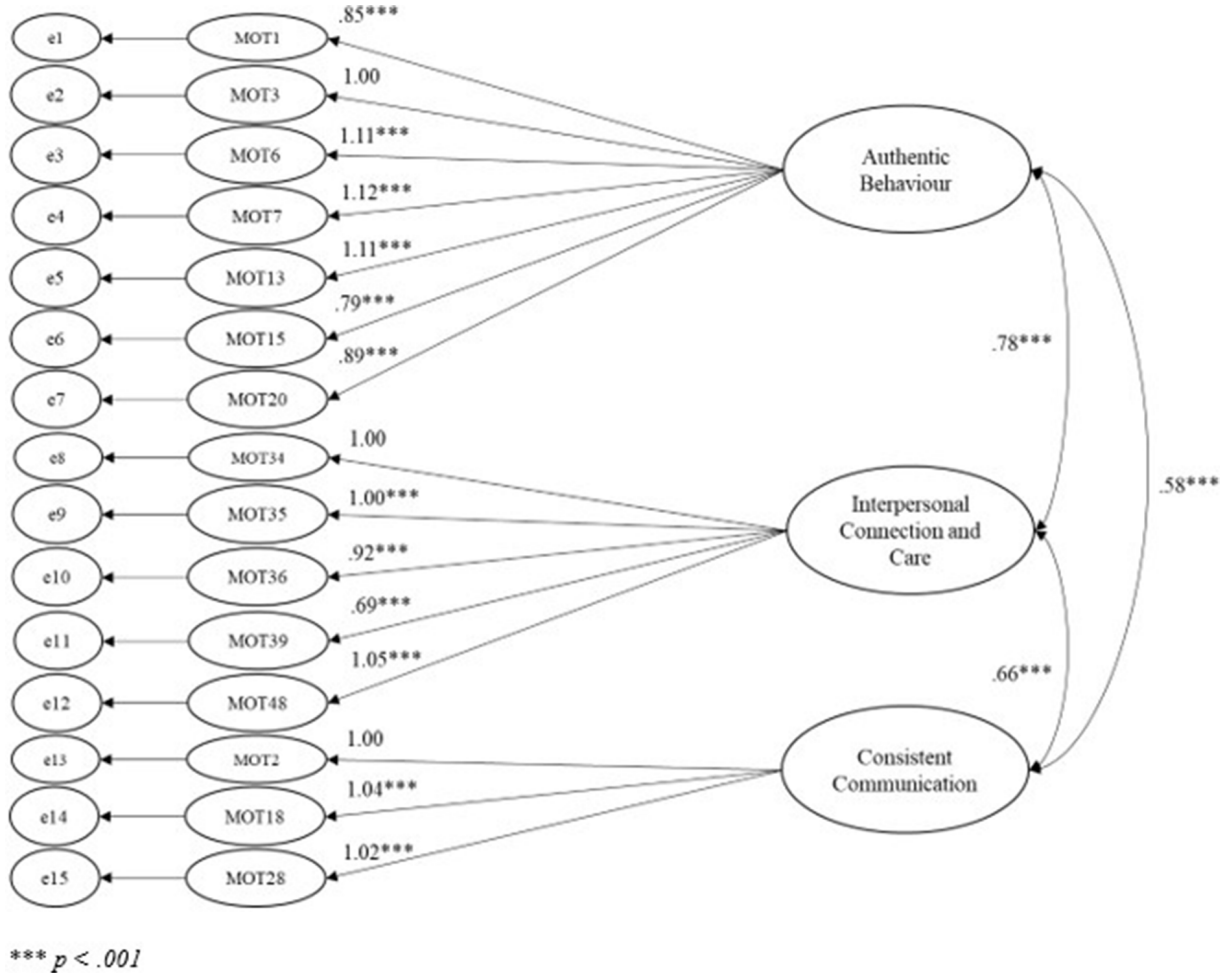
Confirmatory factor analysis model of multidimensional employee trust in leaders.

The results showed a good fit to the model (see Figure 1) based on a range of indices (see Table 2 for interpretation), χ^2^ (87, N = 244) = 171.56, p < 0.001; PCMIN/DF = 1.97; NFI = 0.95; CFI = 0.97; RFI = 0.94; TLI = 0.96; RMSEA = 0.06 and RMSEA confidence interval = 0.05–0.08; SRMR = 0.04. While the χ^2^ result was significant, the χ^2^ statistic is sensitive to sample size and is no longer relied upon as the main source for acceptance or rejection of a model (Schermelleh-Engel et al., 2003; Vandenberg, 2006). Rather, the χ^2^ significant result gives cause to refer to additional fit indices, which is why the full range of reported fit indices were explored. In addition, the model showed excellent fit after the initial item and factor analyses and no further model manipulation was required. Therefore, *H2* was supported.

## Discussion

The aims of these studies were to examine the validity of McAllister’s (1995) Affective and Cognitive Trust scale in the contemporary work environment (*H1*; Study 1) and develop and preliminarily validate an alternate measure of employee multidimensional trust in leaders (EMT) appropriate for the blended workplace using appropriate scale design protocols (*H2*; Studies 2 and 3). Study 1’s examination of McAllister’s (1995) Affective and Cognitive Trust scale achieved the first aim. The item analysis showed that three of the items (3, 5, 11) had low inter-item correlations, well below what would be considered acceptable for a well-validated scale (DeVellis, 2016; ≤ 0.65). Many of the items (2, 4, 7, 9, 10) also suggested multicollinearity^37^ (inter-item total correlations >0.80). These findings in total suggest that the scale has less than optimal internal consistency. Confirmatory factor analysis reported suboptimal fit of the data to the scale’s structural model. McAllister’s (1995) Affective and Cognitive Trust scale has been used often in trust research (McEvily and Tortoriello, 2011). Given the current findings, there is a case to suggest that McAllister’s (1995) scale may not be ideally suited to the contemporary work environment, or at least not for the type of post COVID workplace studied here, and this may impact future research employing that scale.

This study’s findings give cause for reconsidering employee multidimensional employee trust in leaders measurement. The workplace has evolved substantially since 1995. Generational differences (Twenge, 2010), gender representation (Martin and Phillips, 2007), and the influence of globalisation (Alemanno and Cabedoche, 2011) are changes in the workplace identified in the literature. The work environment has also changed, and been improved, by technology (Andreeva and Kianto, 2012), flexible work practices (Spreitzer et al., 2017) and a focus on employee wellbeing (Guest, 2017). The alternative EMT scale identified this with its reference to the importance of consistent communication, perhaps across distance and mediums, and interpersonal caring relationships which links to focussing on wellbeing. Scales measuring complex psychological constructs must be checked for relevancy in a fast-moving society where the work environment may look very different decade by decade.

Studies 2 and 3, a pilot and a preliminary validation of an alternative EMT scale, addressed the second aim. This 15-item scale, with the subscales of Authentic Behaviours, Interpersonal Connection and Care, and Consistent Communication, was shown to have strong psychometric properties. A main outcome of the current series of studies is in regards the multidimensional structure of employee trust in leaders in the contemporary workplace. Consistent with McAllister (1995) and other trust researchers (Dirks and Ferrin, 2002; Schoorman et al., 2007; McEvily and Tortoriello, 2011; Fischer et al., 2020), Study 3 observed a relational dimension and two behavioural dimensions to multidimensional employee trust in leaders. The relational dimension of the alternative EMT scale contains items that describe interpersonal connection and care. This includes vulnerability, sameness, and shared experiences and values. The two behavioural dimensions of the alternative EMT describes (1) authentic behaviours, including maintaining confidentiality, honesty, respect, reliability, and reciprocity and (2) key communication behaviours. The identification of three dimensions in the EMT scale in Study 3 suggests that theory about trust in organisations needs to advance with the evolving workplace context.

Communication has always been essential to organisational performance (Engelbrecht et al., 2017; Fulmer and Ostroff, 2017; Costa et al., 2018), but now it appears to be more important than ever. Although it could be categorised as a behaviour, communication appeared to have a substantial impact on employee trust in leaders in the contemporary work environment and it emerged as its own factor in the analyses. In McAllister’s (1995) Affective and Cognitive Trust scale the construct of ‘communication’ was embedded across the dimensions and their items. This study highlighted the essential element of communication as a unique factor to be measured separately from relationship quality and other trustworthy behaviours. This second emergent behavioural dimension was called “Consistent Communication”. These findings suggest communication is at the core of trust in the contemporary work environment, as employees working physically together and virtually with their teams and leaders is now commonplace.

### Implications

These preliminary findings showed promise for an alternative EMT scale that is applicable to the blended work environment of the contemporary workplace. The behavioural and relational elements of multidimensional employee trust in leaders were upheld, but also showed the significance of communication as its own dimension of trust in the contemporary workplace. This has implications for future trust measurement as previous research has not accounted for three distinct dimensions. McAllister’s (1995) Affective and Cognitive Trust scale only contains two dimensions and does not measure communication as its own dimension. Interpretation of previous multidimensional employee trust in leaders’ literature using McAllister’s (1995) scale must be exercised with caution noting this limitation, and future research should consider measuring all three organisational trust dimensions to be applicable for the contemporary work environment.

The following analyses are proposed to advance the findings of this preliminary analysis. First, discriminant validity analysis is warranted to explore EMT’s association with different constructs, such as job satisfaction or employee engagement. Divergent validity analysis can be used to ensure EMT does not correlate too strongly with measurements of a similar but distinct trait, such as Leader Member Exchange. Construct validity analysis is useful to assess whether a different sample’s EMT CFA results align with the findings of this study. This would also be helpful to replicate the assessment of McAllister’s (1995) Affective and Cognitive Trust scale CFA against the findings of this study with a new sample. Next, predictive validity analysis could be used to determine if EMT predicts an outcome, such as employee engagement or organisational citizenship behaviour. Finally, test–retest reliability analysis should be done to ensure stability in the measure.

### Limitations

Although the findings of this study are promising, there are some limitations. Whilst data collection during the COVID-19 pandemic has been positioned as key to the outcomes of the research, it must also be acknowledged that the experience of forced remote working might have impacted the findings in a way that is less generalisable should remote work become less prominent. Next, while gender was relatively balanced in Study 3, Study 1 had greater male representation and Study 2 had greater female representation. There was a larger sample size of younger employees under the age of 34 than those between 35 and retirement age (approximately 65 and older) in Study 3, while more participants aged 30–39 participated in Studies 1 and 2. Also, there were greater numbers of responses from the Healthcare and Social Assistance and Professional, Scientific and Technical Services than other industries. This potential homogeneity in the population may have influenced the findings in unknown ways and may make the findings more relevant to younger professional adults. Lastly, the use of the two recruitment methods may have influenced the findings. Part of the sample was recruited *via* email, social media, and snowballing through the researcher’s and participants’ communication networks based in Australia. Although the use of social media did improve obtaining responses from outside Australia. The second part of the sample was recruited through a research panel *via* the data collection agency Prolific. Those participants were recruited from a variety of countries. Work location was not asked of participants, so it is not possible to determine if place influenced outcomes. Nevertheless, the potential issue regarding recruitment strategy differences was partially dealt with by exploring comparability through a series of inferential tests and no significant differences between the participant groups were observed.

### Future research

This study has demonstrated the significance of communication as a trustworthy behaviour that requires focus in a blended environment of virtual and face-to-face work contexts. The findings regarding the relationships between the EMT scale dimensions and organisational outcomes indicate important cross-sectional associations and reinforce organisational trust’s multidimensionality. The EMT scale showed three unique dimensions of organisational trust: one relational dimension and two behavioural dimensions. Consistent communication emerged from other trustworthy behaviours as a significant behavioural element of organisational trust. The EMT scale appears to be reliable and valid, but more research is needed to continue to assess the psychometric performance of the scale in other samples.

## Data availability statement

The datasets presented in this article are not readily available because the plain language statement used to invite participation explained that the data will only be accessed by the research team, and that data will be securely stored online within Deakin University and then destroyed after 7 years. This was in the approved ethics application and aligns with the Australian Psychology Society’s Code of Ethics. Requests to access the datasets should be directed to the articles first author, SF, fischerlsarah@gmail.com.

## Ethics statement

The studies involving human participants were reviewed and approved by Deakin University Human Research Ethics Committee Project ID HEAG-H 179_2019. Written informed consent for participation was not required for this study in accordance with the national legislation and the institutional requirements.

## Author contributions

SF designed, conducted, and wrote up the studies into this manuscript. AW and SH supervised SF through this process as part of SF’s PhD. AW was the principal supervisor and contributed to conception and design of the study. SF performed the statistical analysis under the supervision of SH. SF wrote the first draft of the manuscript. All authors contributed to the article and approved the submitted version.

## Conflict of interest

The authors declare that the research was conducted in the absence of any commercial or financial relationships that could be construed as a potential conflict of interest.

## Publisher’s note

All claims expressed in this article are solely those of the authors and do not necessarily represent those of their affiliated organizations, or those of the publisher, the editors and the reviewers. Any product that may be evaluated in this article, or claim that may be made by its manufacturer, is not guaranteed or endorsed by the publisher.
